# A Closer Look at Precision Hard Turning of AISI4340: Multi-Objective Optimization for Simultaneous Low Surface Roughness and High Productivity

**DOI:** 10.3390/ma15062106

**Published:** 2022-03-12

**Authors:** Adel T. Abbas, Abdulhamid A. Al-Abduljabbar, Ibrahim A. Alnaser, Mohamed F. Aly, Islam H. Abdelgaliel, Ahmed Elkaseer

**Affiliations:** 1 Department of Mechanical Engineering, College of Engineering, King Saud University, Riyadh 11421, Saudi Arabia; ajabbar@ksu.edu.sa (A.A.A.-A.); ianaser@ksu.edu.sa (I.A.A.); 2 Department of Mechanical Engineering, School of Sciences and Engineering, The American University in Cairo, AUC Avenue, New Cairo 11835, Egypt; mfawzyaly@aucegypt.edu (M.F.A.); ihamdys@aucegypt.edu (I.H.A.); 3 Department of Mechanical Engineering, Faculty of Engineering, Fayoum University, Fayoum 63514, Egypt; 4 Department of Production Engineering and Mechanical Design, Faculty of Engineering, Port Said University, Port Fouad 42526, Egypt; ahmed.elkaseer@kit.edu; 5 Institute for Automation and Applied Informatics, Karlsruhe Institute of Technology, 76344 Karlsruhe, Germany

**Keywords:** multi-objective optimization, AISI 4340 alloy steel, wiper insert, surface quality, surface integrity, material removal rate

## Abstract

This article reports an extended investigation into the precision hard turning of AISI 4340 alloy steel when machined by two different types of inserts: wiper nose and conventional round nose. It provides a closer look at previously published work and aims at determining the optimal process parameters for simultaneously minimizing surface roughness and maximizing productivity. In the mathematical models developed by the authors, surface roughness at different cutting speeds, depths of cut and feed rates is treated as the objective function. Three robust multi-objective techniques, (1) multi-objective genetic algorithm (MOGA), (2) multi-objective Pareto search algorithm (MOPSA) and (3) multi-objective emperor penguin colony algorithm (MOEPCA), were used to determine the optimal turning parameters when either the wiper or the conventional insert is used, and the results were experimentally validated. To investigate the practicality of the optimization algorithms, two turning scenarios were used. These were the machining of the combustion chamber of a gun barrel, first with an average roughness (Ra) of 0.4 µm and then with 0.8 µm, under conditions of high productivity. In terms of the simultaneous achievement of both high surface quality and productivity in precision hard turning of AISI 4340 alloy steel, this work illustrates that MOPSA provides the best optimal solution for the wiper insert case, and MOEPCA results are the best for the conventional insert. Furthermore, the results extracted from Pareto front plots show that the wiper insert is capable of successfully meeting both the requirements of Ra values of 0.4 µm and 0.8 µm and high productivity. However, the conventional insert could not meet the 0.4 µm Ra requirement; the recorded global minimum was Ra = 0.454 µm, which reveals the superiority of the wiper compared to the conventional insert.

## 1. Introduction

Customer demand for higher surface quality and increased productivity arises due to stringent requirements for recent precise and complicated applications [1]. In this regard, the precision hard turning process, with its proven ability to machine difficult-to-cut materials to produce high-precision, high-quality parts with tight tolerances, has become one of the most promising technologies in the manufacturing field [2,3] and is considered a cost-effective alternative to conventional grinding operations [4,5].

In recent years, researchers have devoted numerous efforts to developing simulation models of the hard turning process in order to predict the surface quality, albeit with the omission of the effect on the material removal rate (MRR), and vice versa [6,7,8], taking productivity into consideration. Initially, only the conventional turning operation parameters, namely, the cutting speed $V_{c}$, the feed rate f and the depth of cut $a_{p}$, were investigated and optimized; however, researchers initiated a new approach that involves the insert type and material as a new operation parameter or so-called “running condition”. The introduction of recently manufactured and presented insert materials and geometries has enhanced the tool life and the surface integrity of the final product, but it has also helped researchers to examine a wider range of conventional running parameters [7,9,10]. Moreover, the use of a suitable coolant, even synthetic [11] or natural vegetable oil [12], has been found to be an influential parameter on the final surface quality and tool wear [12].

A closer look at the literature highlights the need for representative models that quantitively describe the effect of process parameters on hard turning. Sales et al. [13] stated that the combination of all running conditions resulted in desirable surface quality and concluded that the excessive temperature generated during the hard turning process negatively affects the surface roughness (Ra). Additionally, the variability in the geometry of the cutting tool insert influences the final quality of the surface [13].

Manufacturers have introduced various innovative inserts to the industrial sector. Wiper inserts with multi-radii noses have gained researchers’ attention. Wiper inserts outperformed the conventional insert with a single-radius nose in aspects of surface quality and productivity. Surprisingly, the wiper insert is capable of achieving a roughness of 0.12 µm [6,9], while the conventional insert type obtains a roughness of 0.447 µm under the same cutting conditions. It is recognized amongst researchers that the feed rate, the insert nose geometry and the interaction between them are the most influential parameters on the hard turning process [6,14,15,16]. In order to investigate the influence of process parameters, a number of investigators have applied response surface modeling (RSM), full quadratic regression and ANOVA analysis [3,5,6,8,17,18]. Of all tested parameters, the feed rate and insert type were found to have the lowest *p*-values [4,6,14,15,19], followed by the cutting speed [20] and depth of cut. In their study on the influence of parameters, Dhar et al. [21] found that machining with a reduced wear and damage insert produced improved surface roughness and allowed higher cutting speeds and feed rates.

Although investigators have clearly studied the effect of each turning parameter on the surface quality, there is a deficiency of the optimization prospects of the models presented in the literature. In [22], particle swarm optimization was used to search for the optimal number of machining passes and for the optimal running parameters of each pass. Then, Pareto search was used to preselect the solution. Furthermore, a genetic search model was developed on an experimental basis in order to obtain the optimal process parameters [20]. Moreover, from the perspective of pre-machining planning, Yellowley and Adey [23] proposed an adaptive control optimization (ACO) approach that can help manufacturers to preselect process parameters in order to increase productivity and avoid tool damage based on tool geometry, workpiece variation and feed rate control. In addition, a deterministic optimization model was proposed to validate the optimal running conditions of the turning process by means of a numerical study. This study presented an online application of a computer-aided manufacturing (CAM) program that provides manufacturing planning engineers with optimal running conditions in order to improve production time and cost per part [24]. Moreover, simulated annealing (SA) and ant colony (AC) optimization models, respectively developed by Wang [25] and Vijayakumar et al. [26], were compared in order to obtain the optimal running cost of a multi-pass hard turning operation. The results accounted for the cutting speed, feed rate and number of roughing and finishing passes [25,26].

Numerous researchers have provided the manufacturing field with applications for optimization that consider the optimal manufacturing time and cost [27,28]. Additionally, a simulated annealing-particle swarm optimization approach revealed that, in a minimum quantity cooling lubricant (MQCL) environment, the optimal parameters of the turning operation of stainless steel were a cutting speed of 375 m/min, depth of cut of 0.2 mm and feed rate of 0.05 rev/mm [29]. In addition, this study showed that the feed rate was the most influential parameter on surface roughness. Further investigation of the optimization of the turning operation was conducted by using a gray relational analysis that is based on an orthogonal array of the Taguchi method. The obtained cutting parameters considering the minimum surface roughness were a cutting speed of 155 m/min, a feed rate of 0.12 mm/rev and a depth of cut of 0.8 mm [30]. Furthermore, an adaptive approach was presented in which a multi-objective genetic algorithm was modified using linear techniques for multidimensional analysis of preference (LINMAP) [31]. The experimental work was carried out based on higher cutting speeds of up to 200 m/min using two different cutting inserts: wiper and conventional inserts. The targeted variable outputs were the minimization of surface roughness and power consumption and the maximization of the material removal rate. In this research, the optimal parameters for machining AISI 4340 alloy were a cutting speed of 196.8 m/min, a depth of cut of 0.93 mm and a feed rate of 0.14 mm/rev, which resulted in 0.419 µm surface roughness and a high material removal rate of 26,131.6 mm^3^/min [31]. These results show promising progress in the high-speed machining of AISI 4340 steel alloys.

Looking at the reviewed literature, one can argue that the variation in optimal solutions of the turning operation through various optimization techniques is confounding. Hence, extended optimization work is presented in this paper via different multi-objective techniques to achieve the best surface quality and productivity. In particular, this work is an extension of the investigation by Adel T. Abbas [6], in which a comparison between wiper and conventional inserts was conducted through a full factorial experimental design. The investigated parameters were the insert type, the cutting speed, the feed rate and the depth of cut. Notably, a flood coolant was used during the experiments. Two mathematical models of the wiper and conventional insert surface roughness were developed in MATLAB by full quadratic regression. The developed equations were tested. The average absolute error of both equations was around 7 to 10%. This encouraged extensive research to optimize process conditions and productivity by introducing the MRR as an objective function in addition to the surface roughness function. Three multi-objective techniques were used in this research to search for the feasible regions of optimal solutions, and the results were experimentally validated via a number of machining trials. Finally, the results helped in identifying the optimal running conditions of an industrial application in order to achieve higher surface quality (low surface roughness, Ra) and productivity (high material removal rate, MRR). Specifically, productivity can be described as achieving a lower manufacturing lead time, while the material removal rate (MRR) is defined as the rate at which volumetric material is removed during the machining process; hence, productivity is highly related to MRR.

The aim of this research study was to provide deeper insights into the relative machining performance of AISI 4340 alloy steel in terms of obtainable surface roughness and productivity (MRR) using wiper and conventional round-nose carbide inserts and to help identify the optimal cutting conditions that lead to a significant increase in the obtainable material removal rate while maintaining high surface quality. Furthermore, this study entailed a comparative assessment of three different multi-objective optimization techniques.

## 2. Materials and Methods

### 2.1. Experimental Work

#### 2.1.1. Materials

The experimental work presented by Adel T. Abbas [6] is presented briefly. The material used in this research is AISI 4340 alloy steel. The chemical composition of the material is presented in Table 1.

The following steps are the heat treatment procedures of this material: austenitized at 900 °C for 5 h, air-cooled, heated at 880 °C for 5 h, quenched in oil, tempered at 590–600 °C for 8 h and air-cooled. The mechanical properties of the used AISI 4340 alloy were evaluated in-house and are presented in Table 2.

#### 2.1.2. Microstructure of Material

For optical microscopy, the samples were prepared according to standard metallographic sample preparation, which includes grinding using SiC sandpaper, then polished using diamond paste of 1.0 and 0.05 µm and finally etched with 5% Nital to reveal the sample’s microstructure.

#### 2.1.3. Machine and Cutting Inserts

The machine used in the experimental work, reported in [6], is the EMCO Concept Turn 45 CNC lathe (EMCO, Salzburg, Austria).

**Figure 1:**
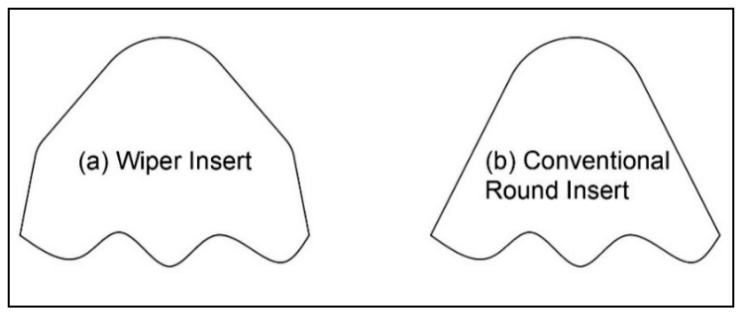
Schematic drawing of the used carbide inserts: (**a**) wiper insert; (**b**) conventional insert.

Two carbide inserts were used for the turning process: wiper insert (DCMX 11 T304-WF GC4325, Figure 1a, Sandvik, Stockholm, Sweden) and conventional round-nose insert (DCMT 11 T304-PF GC4325, Figure 1b, Sandvik, Stockholm, Sweden).

#### 2.1.4. Experimental Trials

All experimental trials were performed using a flood cooling condition (ECO-COOL-MK-3 cutting coolant fluid, Saudi Petroleum Company, Jeddah, Saudi Arabia).

The design of the experiment is a full factorial design of three factors: the cutting speed ($v_{c}$) in m/min, the depth of cut ($a_{p}$) in mm and the feed rate (f) in mm/rev. Each factor is associated with four levels between lower and upper bounds, as shown in Table 3. Hence, this required running the experiment in 128 trials, i.e., 64 for each insert type. The turning trials were conducted by machining cylindrical parts with 50 mm diameter and 130 mm length, in which four segments of 12 mm length were separated by 10 mm clearance.

#### 2.1.5. Surface Roughness Characterization

Finally, the measurement tool used to obtain the surface roughness (Ra) is a Tesa surface roughness tester. A 0.8 mm cut-off length and a measurement speed of 1 mm/s were applied. The Tesa surface tester has a resolution of 0.001 µm. The testing machine conforms to the measurement standards ISO 4287:1997/JIS B0601:2001. The initial surface roughness (Ra) of the workpiece was 1.96 µm on average. In this study, a profile surface parameter, Ra, was used to characterize the generated surface roughness. Future work will entail further analysis of generated surface topography, which can be evaluated by different areal surface parameters [32,33], and thus, deeper insights into the cutting mechanism can be gained.

### 2.2. Mathematical Models

In this stage, after conducting all of the experimental trials, the extracted output was analyzed in MATLAB to develop a full quadratic regression model of the input parameters (independent) and the responses (dependent). Two developed mathematical models were obtained, one for the surface roughness using the wiper insert (see Equation (1)) and the other one for the conventional round insert (see Equation (2)). The subscript (*n*) refers to the normalization of the dependent variables between the lower and upper bounds by the values [–1, 1] (see Equation (3)) [6].

(1) $$\begin{array}{c} {\mathrm{Ra}}_{wiper}=0.518+0.21f_{n}+0.076v_{c}{}_{n}+0.032a_{p}{}_{n}+0.017f_{n}v_{c}{}_{n}-\\ 0.007f_{n}a_{p}{}_{n}-0.008v_{c}{}_{n}a_{p}{}_{n}-0.011f_{n}{}^{2}-0.029v_{c}{{}_{n}}^{2}-0.005a_{p}{{}_{n}}^{2}, \end{array}$$

(2) $$\begin{array}{c} {\mathrm{Ra}}_{conv.}=1.431+0.589f_{n}-0.147v_{c}{}_{n}+0.085a_{p}{}_{n}-0.215f_{n}v_{c}{}_{n}-\\ 0.024f_{n}a_{p}{}_{n}-0.039v_{c}{}_{n}a_{p}{}_{n}-0.026f_{n}{}^{2}-0.127v_{c}{{}_{n}}^{2}-0.017a_{p}{{}_{n}}^{2}, \end{array}$$

(3) $$x_{n}=2\left(\frac{x-x_{min}}{x_{max}-x_{min}}\right)-1.$$

These mathematical quadratic equations were analyzed by ANOVA analysis. It was found that the effects of the parameters on the process were in the order (1) feed rate f, (2) cutting speed $v_{c}$ and (3) depth of cut $a_{p}$ in the case of using the wiper insert, while the conventional insert case had significantly different values in the order (1) feed rate f, (2) the interaction between the feed rate and the cutting speed $f\times v_{c}$ and (3) the cutting speed $v_{c}$. The corresponding *p*-values for each variable in both cases, i.e., wiper and conventional inserts, are shown in Table 4.

### 2.3. Optimization Model Development

Three multi-objective optimization techniques were used on the two types of inserts. In addition to the well-known techniques, a multi-objective genetic algorithm (MOGA), multi-objective Pareto search algorithm (MOPSA) and multi-objective emperor penguin colony algorithm (MOEPCA) were used in this research. MOEPCA is a novel bio-inspired optimizer recently introduced in 2018. In this research [34], MOEPCA was tested via benchmarking functions, and it was compared to well-known robust metaheuristics such as genetic algorithm (GA), particle swarm optimizer (PSO), etc. The proposed optimizer showed higher capabilities of finding low local optima while avoiding false local optima. The new MOEPCA algorithm simulates the emigration movement of penguins and how penguins warm each other while traveling. Hence, this optimizer can be categorized as a radiation-based algorithm. The most beneficial advantage of this algorithm is that it does not face problems with the convergence of parameters as long as the population size is appropriately increased [35]. Not only can MOEPCA solve continuous optimization problems, but it has also evolved to solve binary problems [36] and multi-objective problems [37]. This evolution has helped in solving many problems, including but not limited to those related to cloud service providers and complicated network problems [37].

**Figure 2:**
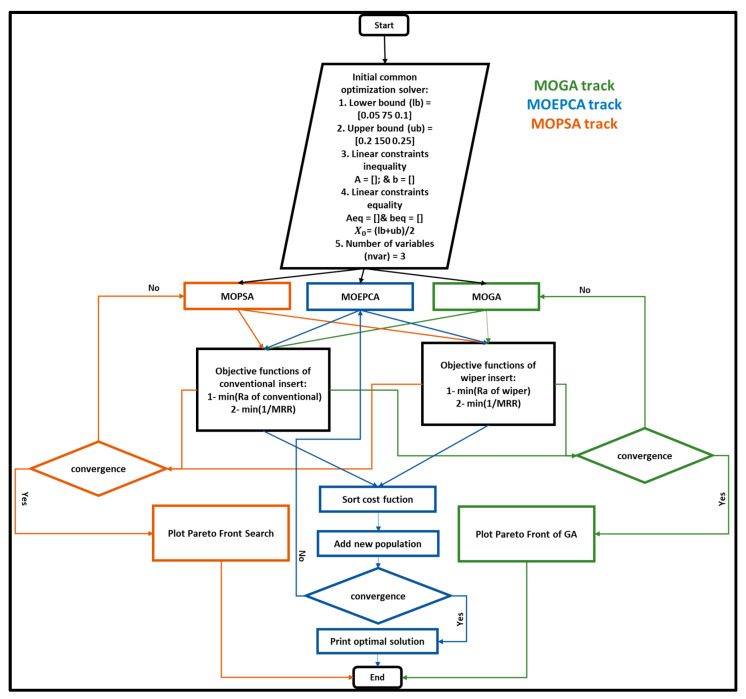
Flowchart of the optimization work.

In this study, the problem was modeled as a non-constrained multi-objective problem. The objective functions of the two cases are a combination of the minimization of surface roughness in Equations (1) and (2) and the maximization of the material removal rate (MRR) in mm^3^/min in Equation (4). The default approach of all optimization techniques is the minimization of the objective function; hence, the minimization of the multiplicative inverse of MRR was taken as an objective. The flowchart of the optimization work is illustrated in Figure 2. The three used optimization techniques started with the same boundary conditions and initial starting points. Additionally, all algorithms were applied to the same objective functions, i.e., minimum surface roughness and maximum material removal rate, as shown in Figure 2. For the MOGA and MOPSA algorithms, the optimization search is dependent on sorted random population generation based on the best parameter set. Meanwhile, MOEPCA relies on a single randomly generated population at the beginning; then, new generated populations are added in the vicinity of the existing population and reordered based on the best cost function score.

(4) $$\mathrm{MRR}=v_{c}fa_{p}$$

The multi-objective mathematical models for both types of inserts are shown in Table 5 below.

## 3. Results and Discussion

### 3.1. Microstructure Analysis

**Figure 3:**
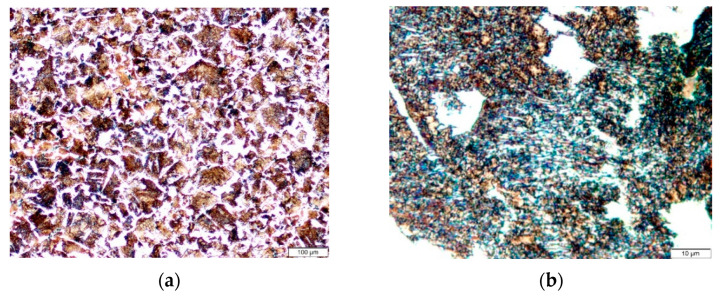
Optical micrograph for AISI 4340 high-strength steel: (**a**) overview microstructure and (**b**) alternate lamellas of ferrite and iron carbide within a pearlite grain.

Figure 3a shows the microstructure of the AISI 4340 material taken by an optical microscope, in which the structure is composed of grains of pearlite (dark) in a matrix of ferrite (light). At higher magnification, Figure 3b shows the alternate lamellas of ferrite and iron carbide within a pearlite grain. Measurements show that the microstructure is composed of nearly 88% pearlite and 12% ferrite, which is the main reason behind the superior strength and hardness of this alloy steel.

### 3.2. Experimental Results

**Figure 4:**
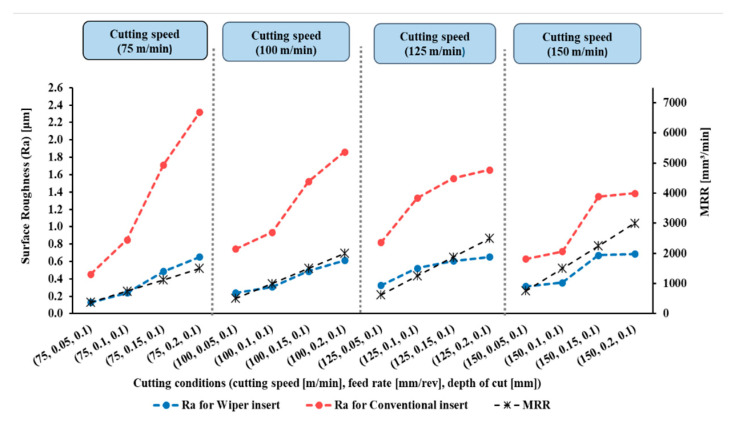
Surface roughness (Ra) and material removal rate (MRR) for AISI 4340 workpieces machined by wiper and conventional inserts at depth of cut (*a_p_*) of 0.10 mm and variable cutting speeds and feed rates (reproduced results originally reported in [6]).

**Figure 5:**
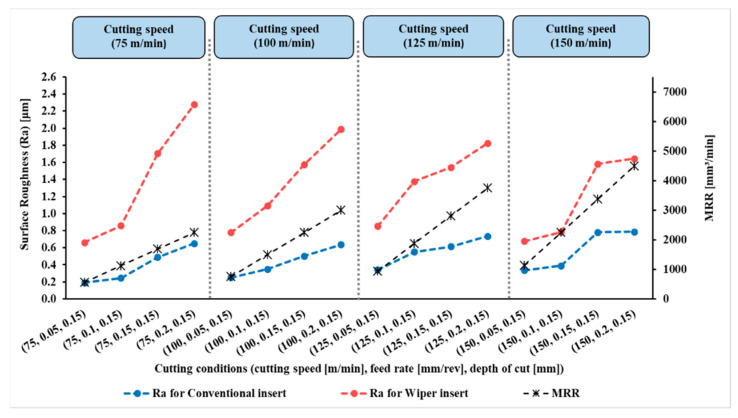
Surface roughness (Ra) and material removal rate (MRR) for AISI 4340 workpieces machined by wiper and conventional inserts at depth of cut (*a_p_*) of 0.15 mm and variable cutting speeds and feed rates (reproduced results originally reported in [6]).

**Figure 6:**
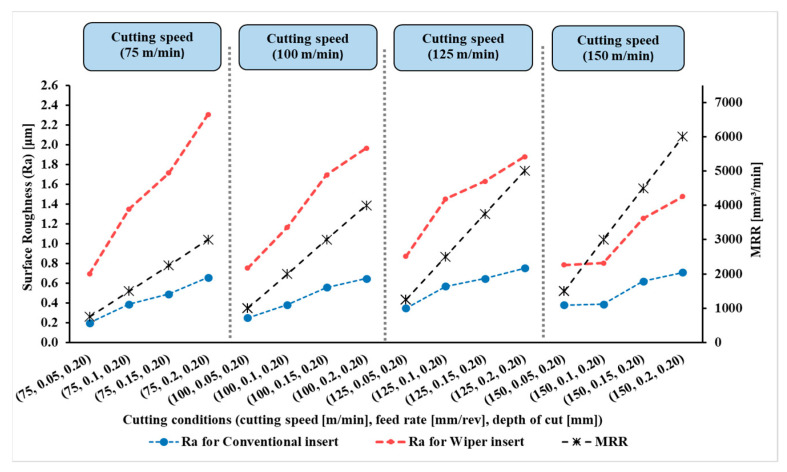
Surface roughness (Ra) and material removal rate (MRR) for AISI 4340 workpieces machined by wiper and conventional inserts at depth of cut (*a_p_*) of 0.20 mm and variant cutting speeds and feed rates (reproduced results originally reported in [6]).

**Figure 7:**
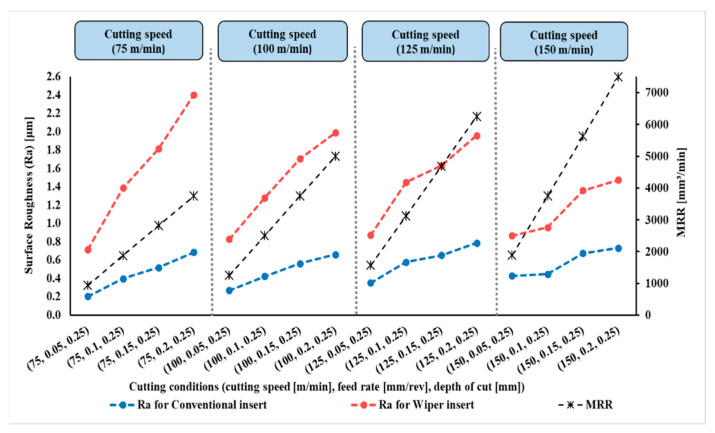
Surface roughness (Ra) and material removal rate (MRR) for AISI 4340 workpieces machined by wiper and conventional inserts at depth of cut (*a_p_*) of 0.25 mm and variable cutting speeds and feed rates (reproduced results originally reported in [6]).

The measured results of surface roughness (Ra) in µm of the 128 experiments were obtained by a Tesa surface roughness tester, as mentioned in the Introduction Section. The results are presented in Figure 4, Figure 5, Figure 6 and Figure 7.

Figure 4, Figure 5, Figure 6 and Figure 7 show the measured values of surface roughness (Ra) and the calculated MRRs for the hard turning of AISI 4340 when machined with wiper and conventional inserts with a range of feed rates and cutting speeds, with depths of cut varying between 0.1 mm and 0.25 mm, respectively. For the whole range of applied parameters, the results show a significant reduction in the resultant surface roughness for the wiper inserts when compared with the conventional ones. For all values of the depth of cut shown in Figure 4, Figure 5, Figure 6 and Figure 7, dramatic proportional trends for the effect of the feed rate on the obtainable surface roughness are observed at low cutting speeds compared to those achieved at higher cutting speeds. The results also reveal that the depth of cut has a considerable influence on the generated surface roughness for all values of feed rate and cutting speed for both wiper and conventional inserts (Figure 4, Figure 5, Figure 6 and Figure 7). With increasing depth of cut, the obtained surface roughness and MRR increased in both cases. The discussion of the full results is thoroughly elaborated in [6].

According to the results, precision hard turning by a wiper insert achieved around 60% improvement compared to using a conventional round insert. The results enabled the development of the mathematical models in Equations (1) and (2). These models were tested under various running conditions that were not included in the experimental trials; however, they lay within the search boundaries. Then, they were tested experimentally. The predicted Ra results from the mathematical model and the measured data from the experiment were compared. It was found that the average absolute errors between predicted and measured outputs were 7% and 10% for the wiper and conventional inserts, respectively. This validation undoubtedly qualifies the mathematical models to be optimized.

### 3.3. Optimization Results

Although the experimental trials covered all possible running conditions between their lower and upper bounds through a full factorial experimental design, the designed levels of factors failed to cover the inter-level parameters. For example, the levels of the feed rate, as mentioned in Table 3, are 0.05, 0.1, 0.15 and 0.2 mm/rev, while the turning machine used in this work is capable of performing 0.005 mm/rev increments. Additionally, for the depth of cut, it can machine the workpiece in 0.01 mm increments. Fortunately, the proposed mathematical model of surface roughness had a low error percentage with respect to the experiment. Hence, an extended investigation through optimization is crucial.

**Figure 8:**
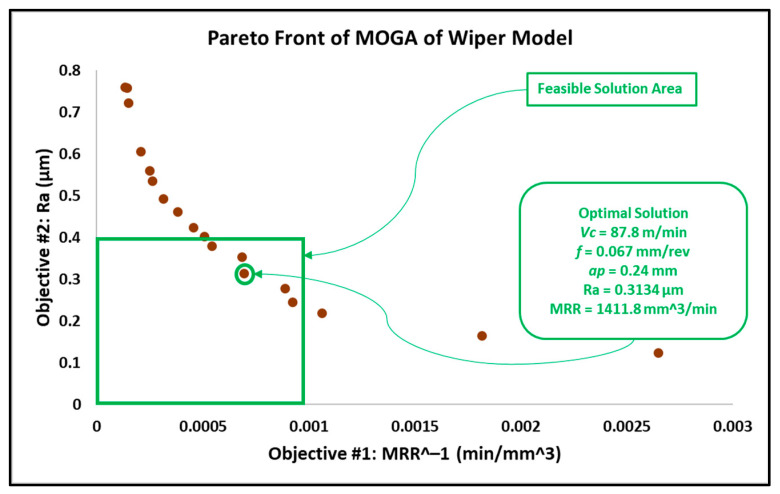
Pareto front plot of MOGA solution of the wiper model.

**Figure 9:**
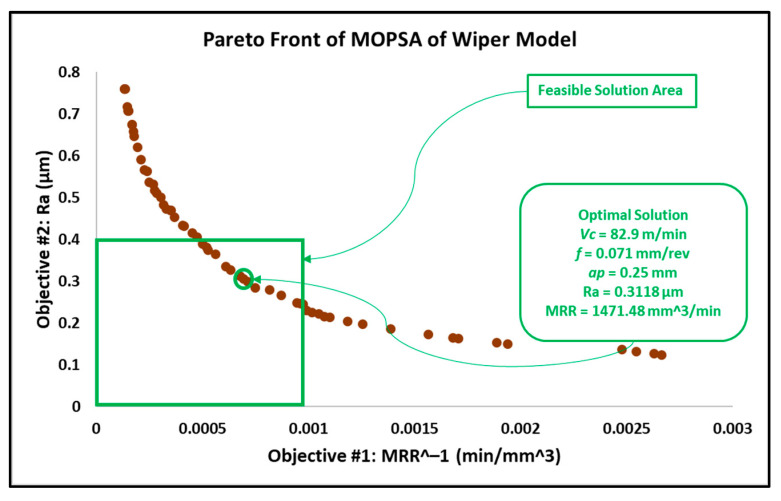
Pareto front plot of MOPSA solution of the wiper model.

**Figure 10:**
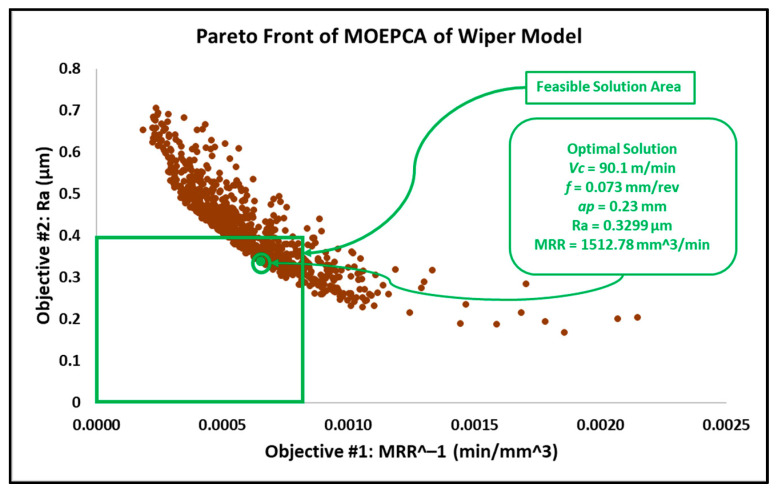
Pareto front plot of MOEPCA solution of the wiper model.

After introducing the MRR as an objective function, the results in this work differed from the results obtained in the previous work reported in [6]. In addition, similar experimental research proposed an adaptive approach via a modified genetic algorithm that searched for the optimal parameters within different search bounds, with a cutting speed between 150 and 200 m/min, a depth of cut between 0.5 and 1 mm and a wider feed rate interval between 0.05 and 0.25 mm/rev [31]. The optimal solution behavior provided by this research is associated with higher cutting speeds and depths of cut, in addition to moderate feed rates. The feasible solution areas of the obtained results from MOGA, MOPSA and MOEPCA are depicted in Figure 8, Figure 9 and Figure 10, respectively.

The optimal solution of all used techniques is recorded in Table 6. The optimal results of MOGA and MOPSA are significantly close, while MOEPCA shows the best optimal results for the MRR but with a slightly higher surface roughness, which is undesirable. However, the obtained results, compared to [31], show moderate levels of cutting speed, low feed rates and higher depths of cut based on the selected parameter levels.

Despite the similar results obtained from MOGA, MOPSA and MOEPCA of the wiper model, MOPSA outperformed the other two algorithms in computational time, as it was 30 times faster than MOGA and 20 times faster than MOEPCA. In addition, the material removal rate obtained from MOEPCA was 3% better than the one obtained from MOPSA. However, MOPSA produced the best optimal solution amongst the three used techniques.

**Figure 11:**
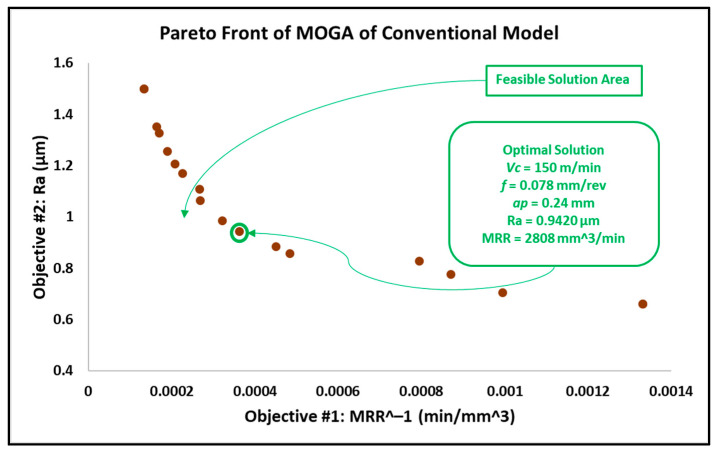
Pareto front plot of MOGA solution of the conventional model.

**Figure 12:**
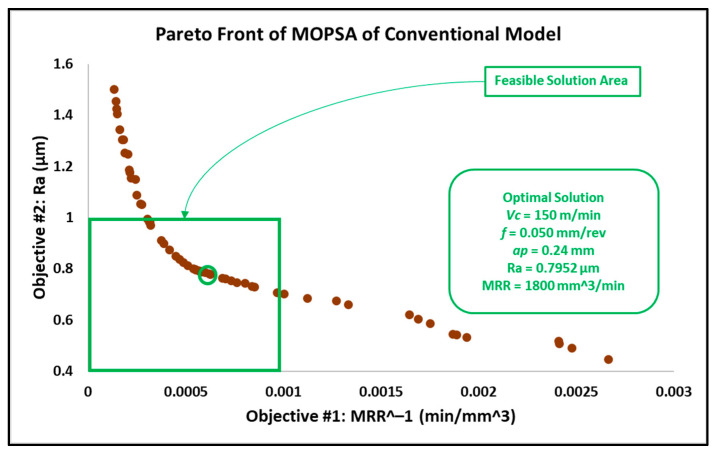
Pareto front plot of MOPSA solution of the conventional model.

**Figure 13:**
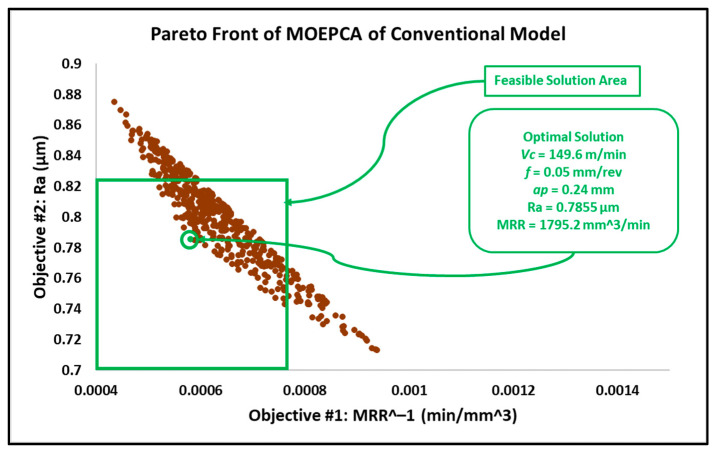
Pareto front plot of MOEPCA solution of the conventional model.

The results of the optimization of the conventional insert model by MOGA, MOPSA and MOEPCA are illustrated in Figure 11, Figure 12 and Figure 13. Additionally, the results of all multi-objective techniques are reported in Table 7.

Taking into account that surface roughness is the first priority, the obtained results from MOPSA and MOEPCA show better surface quality than the MOGA results. However, MOEPCA provided a 1% reduction in surface quality (0.7855 µm to 0.7952 µm). Both MOPSA and MOEPCA provided almost the same MRR (1800 and 1795 mm^3^/min), which is far below those obtained by MOGA (2808 mm^3^/min). Regardless of the fast computational time of MOPSA, the MOEPCA results show the best optimal running conditions.

**Figure 14:**
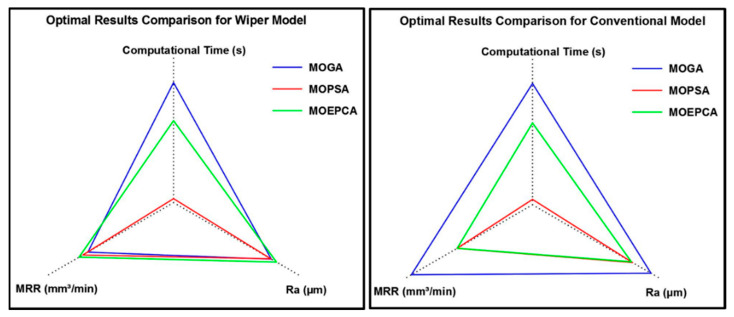
Comparison of optimal results between all optimization techniques for both models: wiper (**left**) and conventional (**right**).

Finally, a comparison between the best optimal results of both models is illustrated in Table 8 and Figure 14.

According to the results, the wiper insert continues to show excellent performance, as mentioned in [6]. The results show that the optimal performance of the wiper insert can produce 252% better surface quality, but with a slightly lower MRR, than optimal conventional round insert running conditions. Figure 14 shows the tradeoff between the resulting surface roughness, the calculated MRR and the computational time of each optimization technique used. MOPSA dramatically outperformed the other techniques for both inserts on the basis of computational time. However, MOEPCA provided a better surface quality in the case of the conventional insert, with a reduction of nearly 2% compared to the Ra obtained from MOPSA.

### 3.4. Experimental Validation and Verification

After identifying the optimal running conditions for the hard precision turning of AISI 4340 stainless steel alloy, experimental trials were conducted in order to validate the obtained results. Each trial with the set of parameters was conducted three times, and the average of the measurements of surface roughness was calculated, as shown in Table 9. 

**Figure 15:**
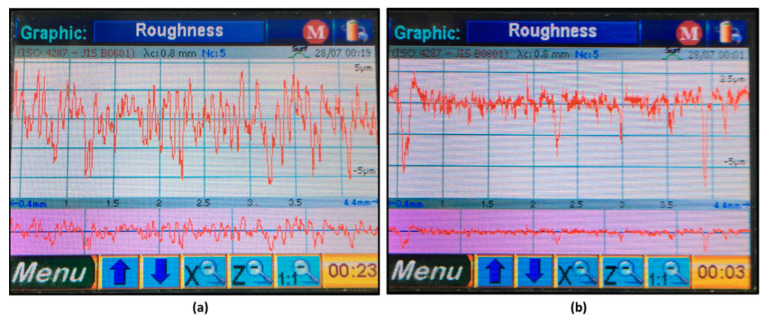
Generated surface roughness of the optimal parameter set for: (**a**) conventional insert and (**b**) wiper insert.

The experimental results of validation and verification match the computed results from the three different algorithms, with a relative error that varies from 5.77% to 12.45%. Such a small range of relative error demonstrates the acceptable accuracy of the optimization algorithms to obtain the proper working conditions for high surface quality and high MRR in high-precision hard turning of AISI 4340 stainless steel alloy. Figure 15 presents examples of the linear surface texture of the machined samples under two optimal sets of running conditions for the conventional insert in Figure 15a and the wiper insert in Figure 15b.

### 3.5. Use Case Applications

As gun barrels are made of AISI 4340 steel alloy, the application chosen as a use case is a combustion chamber of a gun barrel, which is required to be produced at high and critical surface quality. Furthermore, for batch or mass production, higher productivity is required to rapidly complete the work order. Hence, the desired running conditions must satisfy both low surface roughness (Ra) and high material removal rate (MRR). The required surface roughness (Ra) for two combustion chambers is 0.4 µm and 0.8 µm. The results in Section 3.2 in Figure 8, Figure 9, Figure 10, Figure 11, Figure 12 and Figure 13 show that an optimal solution region in the Pareto front plot of MOGA, MOPSA and MOEPCA can be scanned to extract the optimal surface quality and time of the production. The optimal running parameters that lead to higher productivity and surface quality for the two cases are presented in Table 10.

Again, the results indicate that wiper inserts dramatically outperformed conventional round inserts. A surface roughness of 0.4 µm was unachievable with conventional inserts, as previously indicated in the experimental results in [6], which showed that the minimum surface roughness that could be achieved by using the conventional insert was 0.446 µm, whereas the wiper insert was capable of reaching the desired quality. Furthermore, in the case of 0.8 µm surface roughness, the wiper insert could perform the process 4 times faster than the conventional insert, which means higher productivity.

It is worth emphasizing that, based on the obtained optimal process parameters identified, this extended computational investigation provides a closer look at previously published work entailing unrefined experimental results reported in [6]. Furthermore, this computation study successfully determined the optimal process parameters for simultaneously minimizing surface roughness and maximizing productivity in precision hard turning of AISI 4340 alloy steel when machined by two different types of inserts: wiper nose and conventional round nose. The findings of this research study help to identify the optimal cutting conditions, specifically the feed rate, cutting speed and depth of cut when using wiper and conventional inserts, that lead to a significant increase in the obtained material removal rate while maintaining a low resultant surface roughness. One can argue that the results of this research work can assist in obtaining precise, optimal and cost-effective machining solutions that can deliver a high-throughput alternative to conventional grinding when processing difficult-to-cut high-strength AISI 4340 alloy steel in a predictable and controllable manner. In addition, the comparative assessment of three different multi-objective optimization techniques was carried out, and the prime candidate among the evaluated techniques was identified.

## 4. Conclusions

This study involved an extended computational investigation of the experimental work presented in [6] and aimed to improve surface quality and productivity for precision hard turning of AISI 4340 alloy steel using three multi-objective optimization algorithms: MOGA, MOPSA and MOEPCA. The main findings are listed below:

1. The optimal running conditions when using the wiper insert achieved a 252% improvement in surface quality over the results obtained using the conventional round insert, with Ra for wiper insert = 0.3118 µm obtained by MOPSA and Ra for conventional insert = 0.7855 µm obtained by MOEPCA. Meanwhile, the MRR of the conventional insert case was 21.9% higher than that of the wiper insert case.
2. The optimal solutions of the wiper model implemented using the three algorithms were found to be similar with minor differences. MOPSA yielded slightly better results, as it resulted in an MRR of 1471.48 mm^3^/min and Ra of 0.3118 µm at $V_{c}$ = 82.9 m/min, f = 0.071 mm/rev and $a_{p}$ = 0.25 mm compared to the MOGA result, which produced an MRR of 1411.8 mm^3^/min and Ra of 0.3134 µm at $V_{c}$ = 87.8 m/min, f = 0.067 mm/rev and $a_{p}$ = 0.24 mm. However, MOEPCA resulted in a higher MRR of 1512.78 mm^3^/min but a higher surface roughness of 0.3299 µm compared to the other two algorithms.
3. The optimal running conditions of the conventional insert case were obtained by the MOEPCA algorithm at $V_{c}$ = 149.6 m/min, f = 0.050 mm/rev and $a_{p}$ = 0.24 mm with a surface roughness of 0.7855 µm and MRR of 1795.2 mm^3^/min.
4. The conventional insert type showed inferior capability compared to the wiper insert. Unfortunately, the conventional insert type failed to produce acceptable surface roughness (Ra). Moreover, it was outperformed by the wiper insert for producing surface roughness of 0.8 µm, as it yielded an MRR of only 25% of that achieved with the wiper insert.
5. The MOPSA technique outperformed all used techniques in both cases of inserts on the basis of computational time, as it was 30 times faster than MOGA and 20 times faster than MOEPCA.
6. The validation and verification of the proposed models via experimental tests showed that the obtained results of the computational analysis matched the experimental results with a small relative error of 5.77% to 12.45%

## Figures and Tables

**Table 1:** Chemical composition of AISI 4340 [6].

Element	Ni	Cr	Mn	Mo	C	Si	V	Fe
%	2.5	0.9	0.50	0.41	0.40	0.12	0.09	Balance

**Table 2:** Mechanical properties of AISI 4340.

Properties	Value
Ultimate Tensile Strength (MPa)	1200
0.2% Yield Strength (MPa)	1116
Percent Reduction in Area (%)	59
Percent Elongation (%)	9.4
Hardness (HRc)	43.5

**Table 3:** The investigated parameters and their boundaries.

Factor	Levels	Unit
Insert Type	[Wiper, Conventional]	--
Cutting Speed ($V_{c}$)	[75, 100, 125, 150]	m/min
Feed Rate (f)	[0.05, 0.10, 0.15, 0.20]	mm/rev
Depth of Cut ($a_{p}$)	[0.10, 0.15, 0.20, 0.25]	mm

**Table 4:** The calculated *p*-values of variables in ANOVA analysis [6].

Factor	Wiper Insert	Factor	Conventional Insert
f	8.996 × 10^−29^	f	4.351 × 10^−32^
$v_{c}$	8.190 × 10^−11^	$f\times v_{c}$	3.905 × 10^−9^
$a_{p}$	1.520 × 10^−3^	$v_{c}$	3.133 × 10^−8^

**Table 5:** The developed optimization mathematical models of the process.

Model Items	Wiper Model	Conventional Model
Number of Variables	[3]	
Lower Bounds	[0.05, 75, 0.1]	
Upper Bounds	[0.2, 150, 0.25]	
Linear Inequality	[0, 0]	
Linear Equality	[0, 0]	
Initial Starting Point	[0.125, 112.5, 0.175]	
Objective Function #1	Min ($\frac{1}{MRR}$)	
Objective Function #2	Min (${\mathrm{Ra}}_{wiper}$)	Min (${\mathrm{Ra}}_{conv.}$)

**Table 6:** The optimal solution results for the wiper model.

Parameters	Wiper Model		
	MOGA	MOPSA	MOEPCA
f (mm/rev)	0.067	0.071	0.073
$V_{c}$ (m/min)	87.8	82.9	90.1
$a_{p}$ (mm)	0.24	0.25	0.23
Computational time (s)	8.314	0.275	5.67
Ra (µm)	0.3134	0.3118	0.3299
MRR (mm^3^/min)	1411.8	1471.48	1512.78

**Table 7:** The optimal solution results for the conventional model.

Parameters	Conventional Model		
	MOGA	MOPSA	MOEPCA
f (mm/rev)	0.078	0.050	0.050
$V_{c}$ (m/min)	150	150	149.6
$a_{p}$ (mm)	0.24	0.24	0.24
Computational time (s)	8.347	0.37	5.63
Ra (µm)	0.9420	0.7952	0.7855
MRR (mm^3^/min)	2808	1800	1795.2

**Table 8:** Comparison between the best optimal results of both models, wiper and conventional.

Parameters	Wiper Model	Conventional Model
MO Technique	MOPSA	MOEPCA
Computational Time (s)	0.275	5.63
f (mm/rev)	0.071	0.050
$V_{c}$ (m/min)	82.9	149.6
$a_{p}$ (mm)	0.25	0.24
Ra (µm)	0.3118	0.7855
MRR (mm^3^/min)	1471.5	1795.2

**Table 9:** Experiments for validation and verification (VAL/VER) of optimal conditions for wiper and conventional inserts.

Run #	Insert	Software	Speed (m/min)	Feed Rate (mm/rev)	Depth of Cut (mm)	Ra (µm)			Error (%)	MRR (mm^3^/min)
						Software	Experimental			
							Readings	Average		
1	Conventional Insert	MOGA	150	0.078	0.24	0.942	1.072	1.076	12.45%	2808
2							1.08			
3							1.076			
4		MOPSA	150	0.05	0.24	0.7962	0.888	0.88767	10.30%	1800
5							0.892			
6							0.883			
7		MOEPCA	149.6	0.05	0.24	0.7855	0.884	0.884	11.14%	1795.2
8							0.887			
9							0.881			
10	Wiper Insert	MOGA	87.8	0.067	0.24	0.3134	0.342	0.34233	8.45%	1411.8
11							0.34			
12							0.345			
13		MOPSA	82.9	0.071	0.25	0.3119	0.334	0.331	5.77%	1471.5
14							0.331			
15							0.328			
16		MOEPCA	90.1	0.073	0.23	0.3299	0.356	0.35567	7.24%	1512.8
17							0.36			
18							0.351			

**Table 10:** The optimal running conditions for combustion chambers.

Parameters	Ra = 0.4 µm		Ra = 0.8 µm	
	Wiper	Conventional	Wiper	Conventional
f (mm/rev)	0.090	N/A	0.200	0.050
$V_{c}$ (m/min)	87	N/A	150	150
$a_{p}$ (mm)	0.25	N/A	0.25	0.25
Ra (µm)	0.3885	N/A	0.759	0.7989
MRR (mm^3^/min)	1957.5	N/A	7500	1875

N/A means not applicable.

## Data Availability

Not applicable.
